# Contribution of Genome-Wide HCV Genetic Differences to Outcome of Interferon-Based Therapy in Caucasian American and African American Patients

**DOI:** 10.1371/journal.pone.0009032

**Published:** 2010-02-03

**Authors:** Maureen J. Donlin, Nathan A. Cannon, Rajeev Aurora, Jia Li, Abdus S. Wahed, Adrian M. Di Bisceglie, John E. Tavis, for the Virahep-C Study Group

**Affiliations:** 1 Department of Molecular Microbiology and Immunology, Saint Louis University School of Medicine, St. Louis, Missouri, United States of America; 2 Department of Biostatistics, Graduate School of Public Health, University of Pittsburgh, Pittsburgh, Pennsylvania, United States of America; 3 Division of Gastroenterology and Hepatology, Saint Louis University School of Medicine, St. Louis, Missouri, United States of America; 4 Saint Louis University Liver Center, Saint Louis University School of Medicine, St. Louis, Missouri, United States of America; J David Gladstone Institutes, University of California San Francisco, United States of America

## Abstract

**Background:**

Hepatitis C virus (HCV) has six major genotypes, and patients infected with genotype 1 respond less well to interferon-based therapy than other genotypes. African American patients respond to interferon α-based therapy at about half the rate of Caucasian Americans. The effect of HCV's genetic variation on treatment outcome in both racial groups is poorly understood.

**Methodology:**

We determined the near full-length pre-therapy consensus sequences from 94 patients infected with HCV genotype 1a or 1b undergoing treatment with peginterferon α-2a and ribavirin through the Virahep-C study. The sequences were stratified by genotype, race and treatment outcome to identify HCV genetic differences associated with treatment efficacy.

**Principal Findings:**

HCV sequences from patients who achieved sustained viral response were more diverse than sequences from non-responders. These inter-patient diversity differences were found primarily in the NS5A gene in genotype 1a and in core and NS2 in genotype 1b. These differences could not be explained by host selection pressures. Genotype 1b but not 1a African American patients had viral genetic differences that correlated with treatment outcome.

**Conclusions & Significance:**

Higher inter-patient viral genetic diversity correlated with successful treatment, implying that there are HCV genotype 1 strains with intrinsic differences in sensitivity to therapy. Core, NS3 and NS5A have interferon-suppressive activities detectable through *in vitro* assays, and hence these activities also appear to function in human patients. Both preferential infection with relatively resistant HCV variants and host-specific factors appear to contribute to the unusually poor response to therapy in African American patients.

## Introduction

Hepatitis C virus (HCV) causes acute and chronic hepatitis, cirrhosis and hepatocellular carcinoma. Approximately 3.2 million people in the United States are chronically infected with HCV [1]. About 20% of chronically HCV infected patients will develop liver cirrhosis and approximately 10% of those patients will progress to serious decompensated liver disease or hepatocellular carcinoma [2]. HCV is the primary reason for liver transplantation and causes 8000–12,000 deaths each year in the United States [1].

**Figure 1:**
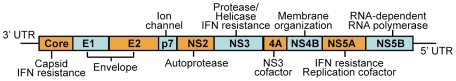
The HCV genome. The HCV genome contains a single major open reading frame flanked by untranslated regions. The 10 genes within the open reading frame are indicated.

HCV is a single-stranded positive polarity RNA virus in the *Flaviviridae* family (reviewed in [3]). It has an open reading frame of ∼9600 nucleotides encoding a polypeptide of ∼3000 amino acids, which is proteolytically cleaved into 10 proteins (Figure 1). HCV is highly diverse genetically, with six major genotypes differing from each other by approximately 30–35% at the nucleotide level. Within each genotype, there may be subtypes that vary by 20–25%, while within each subtype, variation between isolates is typically 10–12% [4].

Treatment for HCV infection employs peginterferon α and ribavirin for 24 to 48 weeks. 50–85% of patients achieve sustained viral response (SVR; undetectable viremia six months post- treatment) depending upon the HCV genotype [5]. Approximately 75% of infections in the United States are with genotype 1 [1] and only 50–60% of patients infected with genotype 1 achieve SVR. The reasons for this poor response are poorly understood [5]. Among genotype 1 infected patients, African-American patients (AA) clear the virus at only about half the rate of Caucasian-Americans (CA) [6]–[8].

The Virahep-C clinical study examined factors associated with non-response to peginterferon α and ribavirin treatment in patients infected with HCV genotype 1a or 1b and found that the poor response to treatment in AA patients was not explained by clinical factors associated with response such as gender, age, obesity, body weight, severity of underlying hepatitis, pre-treatment viral levels, or amount of drug taken [8]. As part of Virahep-C, we examined viral genetic variation in 94 patients and found that higher inter-patient HCV genetic diversity is closely associated with a robust response to therapy at day 28 of treatment. This higher diversity was predominately found in a few genes: NS3 and NS5A in genotype 1a, and core and NS3 in genotype 1b [9]. Importantly, these three genes can counteract the effects of interferon α in cell-based assays (reviewed in [10]). Because day 28 response is driven primarily by response to peginterferon α, these data imply that core, NS3 and NS5A variants with greater diversity are less able to block the effects of interferon *in vivo*, rendering these viruses more susceptible to the very strong interferon response induced by treatment. Our previous analyses examined sequences from patients who had either very poor or very good responses to therapy at day 28. Limiting this analysis to the extremes of the response pattern and measuring the response very early during treatment allowed us to focus on the intrinsic biological effects of the drugs as much as possible. However, the goal of therapy is eradication of the virus, not early suppression of titres, and many biological and non-biological variables can influence response to therapy between day 28 and the six month post-treatment time point at which SVR is defined. Therefore, we asked if there were viral diversity differences associated with SVR, and if so, how they compared to the associations with early response to treatment. To do this, we re-analyzed the Virahep-C HCV sequences when they were stratified by treatment outcome. We also divided the SVR and Non-responder (NR) samples based on the patient race to evaluate whether the HCV genetic associations with response to therapy were similar in the two racial groups.

## Results

### Experimental Design and Patient Selection

The 94 Virahep-C patients that were analyzed in the Virahep-C viral genetics study [9] were re-stratified by genotype (1a or 1b), treatment outcome (SVR or NR) and race (CA or AA). Only the pretreatment sequences were analyzed here; comparisons of the pre- vs. post-treatment genotype 1a sequences are in [11]. The breakdown of the sequences by treatment outcome, patient race and day 28 response is in Table 1. There were similar numbers of SVR and NR patients in the AA and CA groups because the viral genetics cohort was evenly stratified by day 28 response. The baseline clinical characteristics of the patients are in Table 2.

**Table 1:** Cross-tabulation of the number of patients in Virahep-C viral genetics cohort comparing the day 28 response classes and racial groups with treatment outcome.

**Day 28 outcome classes**					
**Genotype**	**Treatment outcome** a	**Marked^b^**	**Poor^c^**	**Intermediate^d^**	**Total**
1a	SVR	15	2	5	22
	NR	1	14	10	25
1b	SVR	13	3	10	26
	NR	2	13	6	21
**Race of patient^e^**					
		**CA**	**AA**	**Total**	
1a	SVR	11	11	22	
	NR	13	12	25	
1b	SVR	16	10	26	
	NR	7	14	21	

a SVR, sustained viral response; NR, non-response.

b Greater than 3.5 log drop in viral titre or to undetectable between baseline and day 28.

c Less than 1.4 log drop in viral titre between baseline and day 28.

d Between 1.4 and 3.5 log drop in viral titre between baseline and day 28.

e CA, Caucasian American; AA, African American.

**Table 2:** Baseline characteristics of patients in the Virahep-C viral genetics cohort.

Feature	Statistic	SVR^a^	NR^b^	P value^c^
Number of patients	N	48	46	–
AA^d^	N (%)	21 (43.8%)	26 (56.5%)	0.2207
Male	N (%)	31 (64.6%)	37 (80.4%)	0.0640
Genotype 1a	N (%)	22 (45.8%)	25 (54.3%)	0.5606
Age (years)	median (25^th^, 75^th^)	46.5 (42.0, 50.0)	49.0 (45.0,53.0)	0.2777
Body weight (kg)	median (25^th^, 75^th^)	84.6 (72.0, 96.8)	89.6 (79.2, 101.7)	0.1022
HCV RNA (log_10_IU/ml)	median (25^th^, 75^th^)	6.0 (5.4, 6.8)	6.6 (6.4, 6.7)	0.0006
ALT^e^ (U/L)	median (25^th^, 75^th^)	60.5 (44.0, 88.0)	69.5 (54.0, 102.0)	0.6145
Albumin (g/dl)	median (25^th^, 75^th^)	4.2 (4.0, 4.4)	4.2 (4.0, 4.4)	0.8317
Platelets (×1,000/mm)	median (25^th^, 75^th^)	249.0 (197.5, 296.0)	205.5 (173.0, 250.0)	0.0006
AFP^f^ (ng/ml)	median (25^th^, 75^th^)	4.1 (2.6, 5.6)	5.6 (3.7, 11.6)	0.7657
Ishak necroinflamatory score (0–18)	median (25^th^, 75^th^)	7.0 (5.0, 9.0)	7.5 (6.0, 9.0)	0.1109
Ishak fibrosis score (0–6)	median (25^th^, 75^th^)	2.0 (1.0, 3.0)	2.0 (1.0, 3.0)	0.2561

a Sustained viral response.

b Non-response.

c Univariate Poisson regression model.

d African American.

e Alanine aminotransferase in serum.

f Alpha fetal protein in serum.

### Use of the HCV Consensus Sequence

HCV replicates as a quasispecies, therefore, the virus can be represented genetically either by characterizing the quasispecies distribution or by using the consensus sequence of the quasispecies to reflect the center of the genetic distribution in each individual. We used the consensus sequence as determined by directly sequencing HCV cDNA amplified from plasma [12] because this study was designed to assess the role of inter-patient genetic variation on outcome of treatment. We have compared the consensus sequence with a near full-length quasispecies analysis in the same patient and found that the consensus sequence was near the center of the quasispecies distribution [13].

### Inter-Patient HCV Genetic Diversity Is Associated with SVR

**Figure 2:**
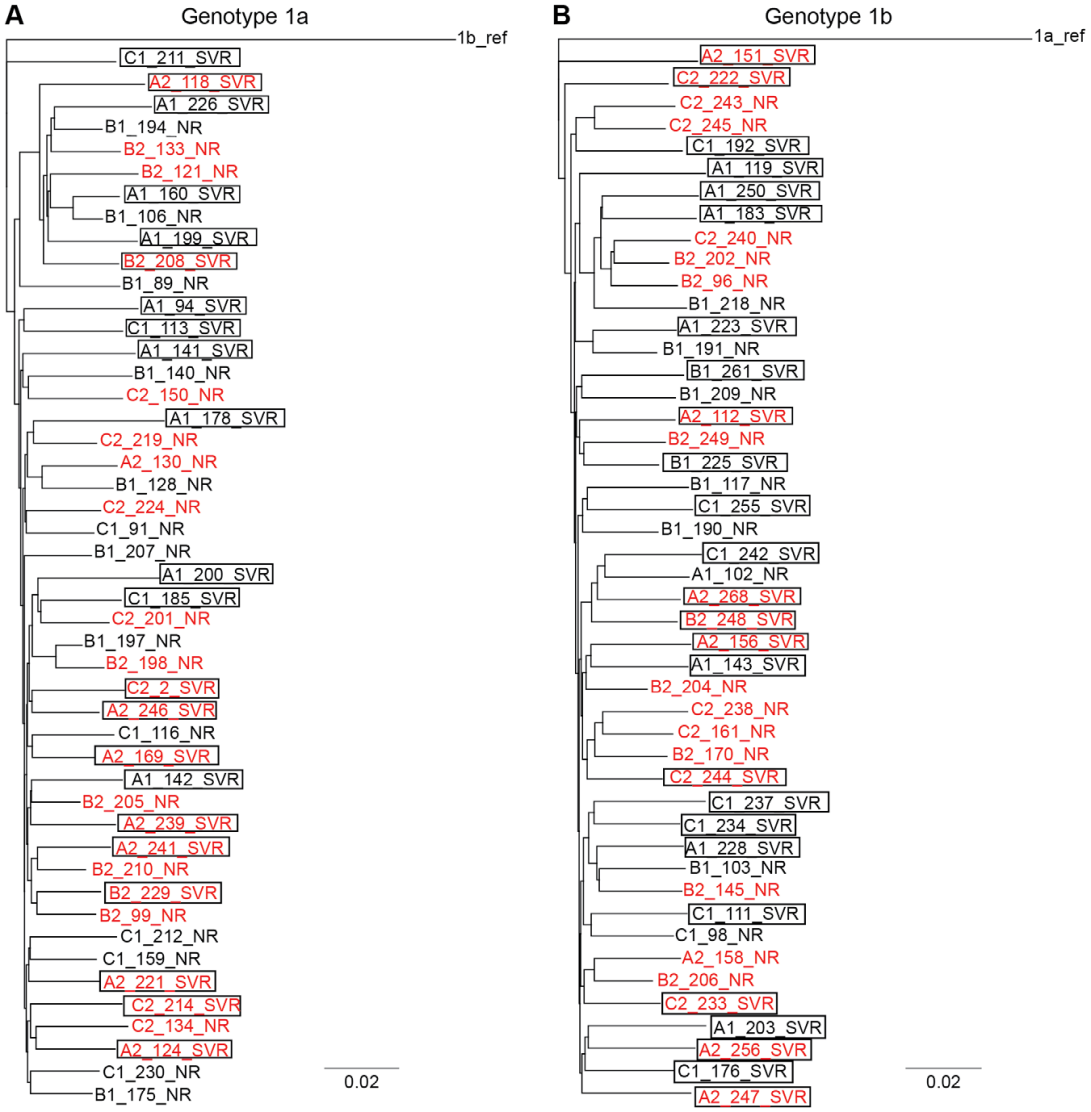
Phylogenetic tree for the genotype 1 polyprotein sequences. (**A**) A maximum-parsimony tree was generated from an alignment of all 47 genotype 1a near full-length polyprotein sequences, with the genotype 1b consensus reference as the out-group. (**B**) A maximum-parsimony tree was generated from an alignment of all 47 genotype 1b near full-length polyprotein sequences, with the genotype 1a consensus reference as the out-group. Treatment outcome is indicated by SVR (boxed names) and NR (not boxed). AA patient sequences are shown in red; CA patient sequences are in black.

An alignment and phylogenetic analysis of all SVR and NR sequences revealed no clustering by response class (Figure 2), and no single or limited number of amino acid variations were closely associated with treatment outcome (data not shown). These results have two implications. First, they indicate that the viruses from the SVR and NR patients are not derived from separate evolutionary lineages. Second, they reveal that response to interferon α-based therapy is not strongly dependent upon simple amino acid variations, such as are seen with antiviral therapies which target the active site of a viral enzyme. These observations are consistent with the pleoitropic cellular effects induced by interferon α, and they imply that viral genetic signatures associated with response to interferon-based antiviral therapy would be spread diffusely through the viral genome. Therefore, we characterized the HCV genetic diversity patterns in the pre-therapy genomes. This analysis differed from our previous analysis [9] in that (*i*) stratification was by treatment outcome (SVR vs NR) rather than day 28 response, (*ii*) inclusion of the 30 day 28 intermediate responders who were excluded from most of our previous analyses to focus on the extremes of early response to treatment, and (*iii*) performance of additional analyses to evaluate the association of genetic variability with treatment outcome.

**Figure 3:**
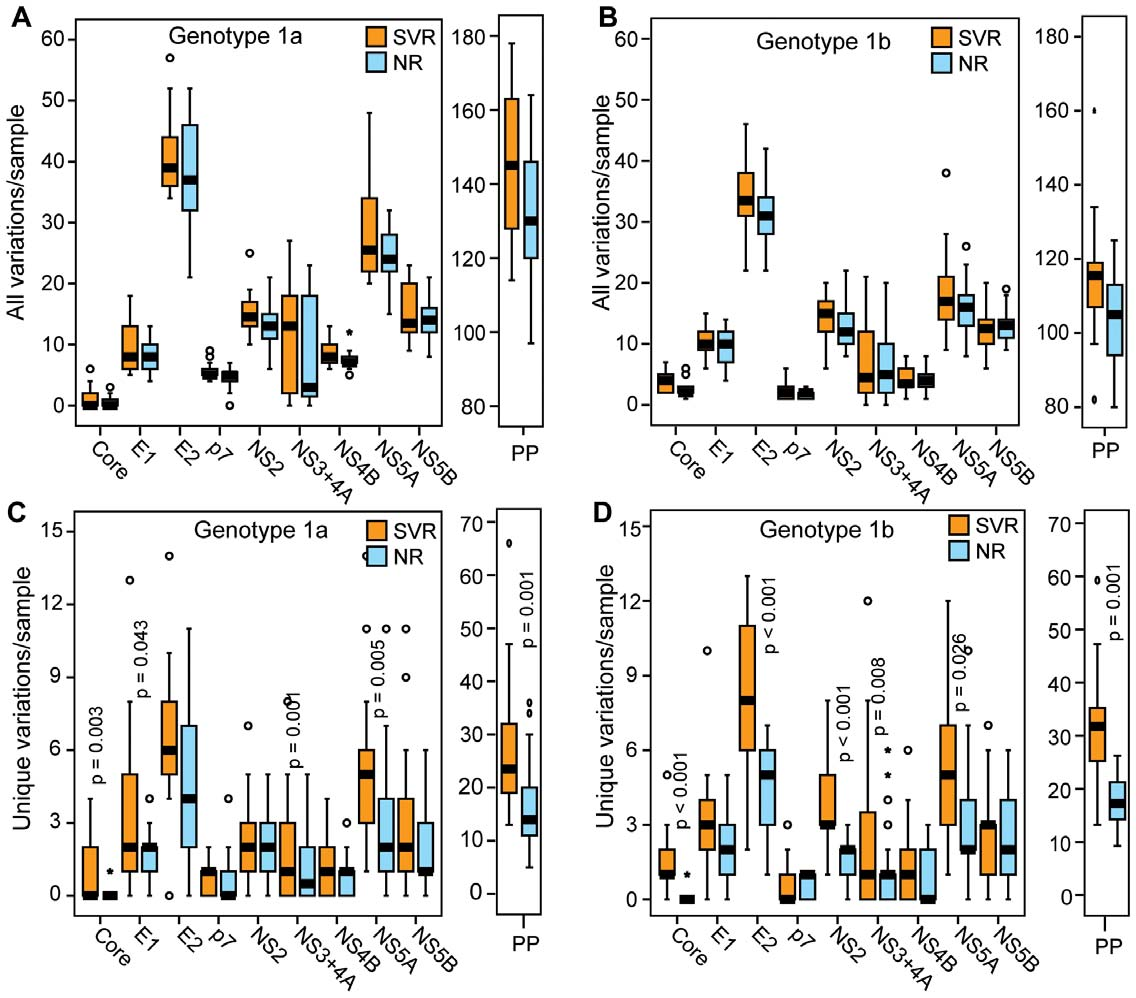
Number of variations per sample by treatment outcome. The number of variations relative to a population consensus is shown for the polyprotein (PP) and for each gene within the polyprotein. Statistical significance is shown for genes with p≤0.05. (**A**) Genotype 1a, all variations. (**B**) Genotype 1b, all variations. (**C**) Genotype 1a, variations unique to the SVR or NR classes. (**D**) Genotype 1b, variations unique to the SVR or NR classes.

We first compared the number of variations in the SVR or NR sequences relative to a genotype 1a or 1b population consensus reference sequence. This reference sequence represents an “average” HCV sequence that is largely free from patient-specific adaptations, such as those that can be driven by T-cell mediated pressures [14]. Overall, the SVR sequences tended to be more diverse, but these differences were not statistically significant (Figures 3A and 3B). However, most HCV genetic variations are anticipated to be neutral, so we eliminated the variations that were common to both the SVR and NR sequences to focus on the subset of variations which were most strongly associated with response. The number of variations that was unique to the SVR sequences was significantly higher than the number unique to NR sequences for the entire polyprotein in both genotypes 1a and 1b (p  =  0.0010; Figures 3C and 3D). When the individual viral genes were assessed, these differences were statistically significant at the p≤0.05 level for genotype 1a core, E1, NS3/4A and NS5A. For genotype 1b, the differences were significant for core, E2, NS2, NS3/4A and NS5A.

As a second measure of diversity, we analyzed the proportion of unique variations relative to the total number of variations in the SVR and NR sequences. For both genotypes 1a and 1b, there were significantly more unique variations than expected in the polyprotein of SVR patients and fewer unique than expected in the NR sequences (p<0.001 for both, Table S1). For genotype 1a, a significantly higher than expected proportion of unique variations was observed for E1, E2, NS3/4A, NS5A and NS5B. For genotype 1b, the core, E2, NS2, NS3/4A, NS4B and NS5A proteins had significantly more unique variations than expected.

The previous measures of inter-patient HCV diversity can be influenced by variations in the external reference sequence, although our use of a population-wide consensus sequence dampens this concern relative to using an arbitrary isolate as a reference. Therefore, we assessed the genetic variation among the HCV sequences by two additional measures that are not dependent upon an external reference sequence, genetic distance and Shannon's entropy. Differences in the genetic distance among the SVR and NR sequences were identified by determining the genetic distance between each pair of sequences and then comparing the average genetic distances between the two classes. p7 was not analyzed by this method because it is so short that small differences are magnified in the genetic distance calculations. In both genotypes 1a and 1b, the average pairwise genetic distance of the SVR polyprotein sequences was significantly larger than the NR distance (Figures S1A and S1B). The genetic distances of the NS3/4A and NS5A SVR sequences were significantly higher for genotype 1a. The genotype 1b core, NS2 and NS3/4A genes from the SVR sequences had significantly higher average genetic distance compared to the NR sequences, with E2 approaching significance (p = 0.052). Finally, Shannon's entropy for each position in alignments of SVR and NR sequences was assessed. When the entire polyprotein was examined, the entropy of the SVR samples was significantly higher than for the NR samples for both genotypes (data not shown). For genotype 1a, core and NS5A had significantly higher entropy for the SVR sequences compared to NR sequences (Figure S2A). For genotype 1b, the entropy values for core were significantly higher for SVR sequences (Figure S2B).

In summary, the SVR polyprotein sequences were significantly more diverse than the NR sequences by all four analytical methods in both genotypes. For genotype 1a, NS5A was significantly more diverse in the SVR samples by all four measures and core, E1 and NS3/4A were more diverse by two of the measures. For genotype 1b, core was more diverse in the SVR samples by all measures, and E2, NS2, NS3/4A and NS5A were more diverse in the SVR samples by two or three of the four measures.

### Effects of Host Responses on Viral Diversity

**Figure 4:**
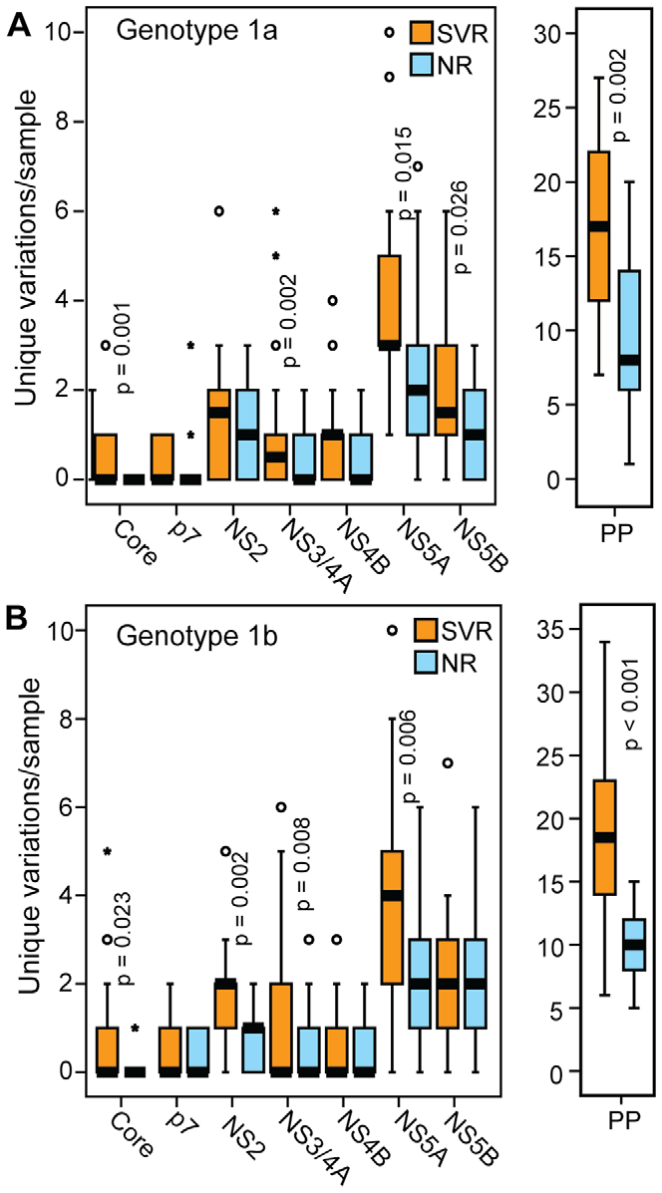
Number of unique variations in non-epitope regions by treatment outcome. Variations unique to the SVR or NR sequences that were not in any known or predicted T-cell epitope were compared in the SVR and NR samples. Statistical significance is indicated where p≤0.05. (**A**) Genotype 1a. (**B**) Genotype 1b.

These diversity differences may be due to pre-existing viral genetic differences that are causally associated with response. Alternatively, patients who respond to therapy may have mounted more effective T-cell responses, which could have driven greater immune escape. This hypothesis is supported by the elevated HCV-specific T-cell immune response among Virahep-C SVR patients compared to NR patients [15]. To evaluate diversity patterns in regions of the viral genome that were unlikely to be under immune selection, we eliminated all known or predicted T-cell epitope sequences [16] from this analysis regardless of their restriction profile because the human lymphocyte antigen identities were not available for these patients. We also eliminated the E1 and E2 proteins because they are under humoral immune selection. This approach eliminates far more sequence for each HCV genome than is actually under immune selection in a given patient, but its advantage is that the remaining sequences are very unlikely to be under substantial immune selection. The mean number of unique variations per sample *outside* T-cell epitopes was compared between the response groups using a Poisson regression analysis. For genotype 1a, diversity differences between the SVR and NR sequences in non-epitope sequences were significant for the polyprotein, core, NS3/4A, NS5A and NS5B (Figure 4A). For genotype 1b, the diversity differences were significant for the polyprotein, core, NS2, NS3/4A and NS5A when the epitope sequences were excluded (Figure 4B). Therefore, immune selection could not account for all diversity differences between the SVR and NR sequences.

**Figure 5:**
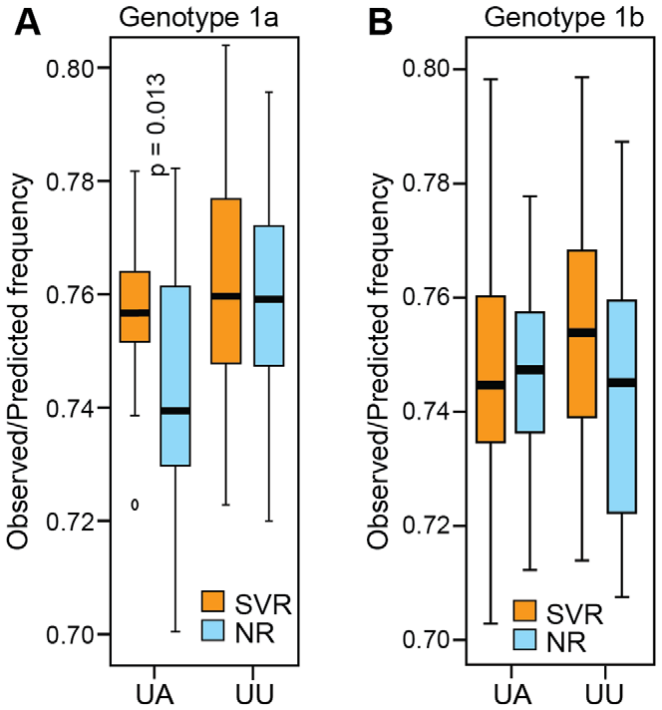
Ratio of observed to predicted UU and UA dinucleotide frequencies. The ratios of observed to predicted frequency of the dinucleotides UU and UA across the polyprotein were compared between SVR and NR patients. Statistical significance is indicated where p≤0.05. (**A**) Genotype 1a. (**B**) Genotype 1b.

We next examined the observed/predicted ratio of UA and UU dinucleotide frequencies in each sequence because these dinucleotides are substrates for RNaseL, a major effecter of the type 1 interferon response. The observed/predicted UA and UU ratios were less than 1.0 in all cases (Figure 5). For genotype 1a, the observed/predicted UA ratio was higher in the SVR than NR sequences (p = 0.013), but the UU ratio was the same in the SVR and NR sequences. No significant differences either the UU or UA ratios for genotype 1B were observed. This implies that all of the sequences were under negative selective pressure from RNaseL, but that the SVR and NR sequences were under similar levels of pressure.

Recent work has identified eight cellular micro RNAs (miRNA) which are induced by interferon β and have potential binding sites in the HCV genome; some of these miRNAs have anti-viral activity against HCV in cell culture [17]. The open reading frame for each HCV genome in this study was examined for possible miRNA targets for these eight miRNAs as well as miRNA-122, a liver-specific miRNA the promotes HCV replication [18]. The frequency of each match was determined for the SVR and NR sequences as a group as well as for each sequence individually. There were no significant differences in the frequencies of any single miRNA seed match between SVR and NR sequences (data not shown). The miRNAs were then grouped according to whether they demonstrated anti-viral activity in culture or not. No differences between NR and SVR were apparent in the frequency with which the different groups of miRNA binding sites appear in the genomes (Figure S3). In addition, the frequency of miRNA-122 sites in the genomes also did not differ between the SVR and NR sequences (data not shown). Therefore, if IFN-stimulated miRNAs regulate HCV's response to treatment, both response classes appear to be equally susceptible to that regulation. Note that this conclusion is limited to the HCV ORF because 3′ or 5′ UTR sequences were not available for all samples.

### Prediction of Treatment Outcome Based on Amino Acid Variations Unique to Day 28 Response

We previously reported that HCV sequences from day 28 marked responders are more variable than sequences from poor responders [9]. All patients in the Virahep-C viral genetics cohort had perfect drug compliance until day 28, therefore, HCV genetic variations associated with day 28 response are related to the intrinsic biological sensitivity of the virus in these patients to therapy, without confounding issues that are present during the demanding 24–48 week treatment regimen. We therefore assessed whether pretreatment genetic variability associated with day 28 response could predict SVR. Table 3 shows the relative risks of achieving SVR based on the number of variations unique to the marked/poor day 28 response classes per genome. In both genotypes, higher numbers of marked/poor unique variations in the polyprotein were associated with a significantly higher probability of SVR. For each additional day 28 response-specific unique variation, the probability of SVR was increased by 3% in genotype 1a and 2% in genotype 1b. When each gene was analyzed separately, core, E1, E2, NS3/4A, NS4B, NS5A and NS5B were significant in genotype 1a, and core, E2, NS2 and NS3/4A were significant for genotype 1b, with up to a 28% increase in the probability of achieving SVR per additional unique variation (genotype 1b core, Table 3).

**Table 3:** Quantitative effect of each additional HCV genetic variation that is unique to the marked or poor day 28 response classes on eventual treatment outcomea.

	Genotype 1a		Genotype 1b	
Protein	Relative Risk^b^ (95% CI^c^)	P value^d^	Relative Risk^b^ (95% CI^c^)	P value^d^
Polyprotein	1.03 (1.02, 1.04)	<0.0001	1.02 (1.01, 1.04)	0.0019
Core	1.26 (1.11, 1.44)	0.0005	1.28 (1.14, 1.44)	<0.0001
E1	1.18 (1.07, 1.30)	0.0006	1.03 (0.88, 1.20)	0.7243
E2	1.10 (1.04, 1.17)	0.0012	1.08 (1.02, 1.15)	0.0102
p7	1.26 (0.87, 1.83)	0.2245	1.05 (0.74, 1.47)	0.7874
NS2	1.13 (0.99, 1.29)	0.0816	1.11 (1.04, 1.19)	0.0023
NS3+NS4A	1.14 (1.05, 1.25)	0.0029	1.08 (1.01, 1.16)	0.0232
NS4B	1.26 (1.01, 1.57)	0.0386	1.08 (0.92, 1.28)	0.3311
NS5A	1.06 (1.03, 1.10)	0.0005	1.03 (0.99, 1.07)	0.1127
NS5B	1.19 (1.08, 1.31)	0.0004	0.95 (0.82, 1.10)	0.4926

a Comparing SVR (sustained viral response) vs. NR (non-response).

b For example, a relative risk of 1.18 for E1 in genotype 1a indicates that for one additional marked/poor response-specific unique variation in this gene, the probability of SVR is increased by 18%.

c CI, confidence interval.

d Poisson regression analysis.

### Effect of Patient Race on Viral Diversity Associated with SVR

The lower response of AA compared to CA patients in the Virahep-C study could not be explained by disease characteristics, baseline viral levels or amount of medication taken [8], and we observed few significant effects of race on associations of inter-patient HCV genetic diversity with day 28 outcome [9]. However, day 28 response is primarily interferon-driven, whereas ribavirin has a major impact on SVR rates [5], [19]. Furthermore, the antiviral mechanisms that suppress HCV titres may change after day 28. Therefore, we asked whether the associations of viral genetic diversity with SVR or NR differed between CA and AA patients.

We first aligned the 1a and 1b sequences and generated a neighbor-joining tree, using a different subtype consensus reference as an out-group. There was no phylogenetic clustering by race for either genotype 1a (Figure 2a) or genotype 1b (Figure 2b). This indicates that the CA and AA patients are not infected with substantially different HCV strains.

**Figure 6:**
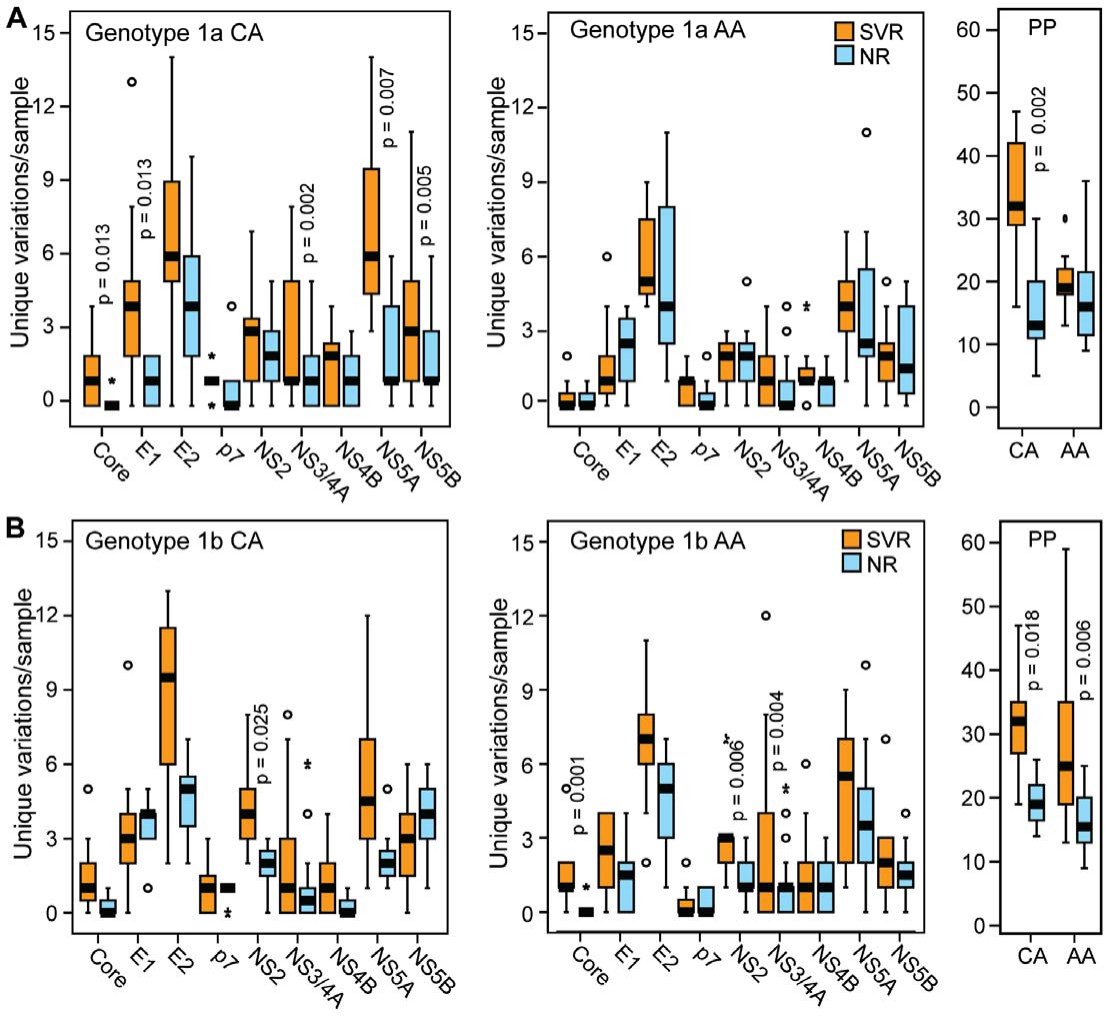
Number of unique variations per sample by treatment outcome and patient race. The number of variations unique to either the SVR or NR groups is shown for the polyprotein (PP) and for each HCV gene for CA and AA patients. Statistical significance is shown for genes with p≤0.05. (**A**) Genotype 1a. (**B**) Genotype 1b.

We next evaluated HCV genetic diversity differences in the CA and AA patients by separating the data in Figure 3 by race of the patient. For genotype 1a, there were significantly more unique variations in the SVR group compared to the NR sequences for the polyprotein, core, E1, p7, NS3/4A and NS5A in the CA patients, whereas no significant differences were observed for AA patients (Figure 6A). For 1b, the diversity differences between the SVR and NR sequences in the polyprotein, core, E2, NS2 and NS5A in the CA patients (Figure 6B). Within genotype 1b AA patients, the diversity differences between the SVR and NR sequences were also significant for the polyprotein, core and NS2 (Figure 6B).

Overall, splitting the data by race of the patient reduced the statistical power of the analysis, but the diversity patterns observed in the combined data sets were largely still apparent in the genotype 1b CA and AA patients and the genotype 1a CA patients. However, these patterns were essentially absent in the genotype 1a AA patients. This difference was most obvious when the full polyprotein sequences were compared (Figure 6).

## Discussion

The major HCV genotypes differ in their response to therapy [5], [19], but the effects of HCV genetic variation within a genotype on treatment outcome are poorly understood. Here, we found pre-treatment genetic variation was robustly associated with outcome of treatment in the two major subtypes of genotype 1. Immune selection could not account for all of the genetic association (Figure 4), and we found no evidence for differential RNaseL cleavage [20] (Figure 5) or differential regulation by cellular miRNAs [17] (Figure S3) of the sequences in the SVR or NR groups. Therefore, much of the difference in the pre-treatment inter-patient viral genetic diversity appears to be causally associated with treatment outcome.

The most consistent viral genetic associations with treatment outcome were found in NS5A for genotype 1a and in core and NS2 for genotype 1b. Our previous analysis comparing the day 28 marked and poor responders in a subset of the same cohort revealed higher inter-patient diversity in the marked responders in NS3 and NS5A for genotype 1a and in the core and NS3 for genotype 1b [9]. Higher diversity was therefore associated with worse response to the drugs for both day 28 response and treatment outcome in the 1a NS5A and 1b core genes. The association of higher diversity in NS2 with SVR for genotype 1b but not with day 28 response may be due in part to increased statistical power associated with the greater number of samples in the treatment outcome analyses. The reduced strength of the association of NS3 with treatment outcome compared to the day 28 response may be due to patient variables (such as drug tolerance) influencing the outcome more over the longer time period, and/or that the treatment outcome associations may be influenced by both interferon α and ribavirin, whereas day 28 response is primarily influenced by interferon α.

The robust association of genetic diversity in core, NS3/4A and NS5A with response to therapy strongly suggests that the mechanisms by which genetic diversity affects treatment efficacy are through altering the interferon-suppressive activities that have been identified for these proteins *in vitro* [21]–[23]. The similarity of the genetic associations with day 28 response and treatment outcome suggests that the roles of these activities remain relatively constant throughout the course of therapy. The distribution of higher diversity associated with suppression of viral titers across multiple viral proteins suggests that there are many ways lesions to the HCV genome could reduce the efficacy of these suppressive functions. HCV's multi-pronged approach to controlling the type 1 interferon response may explain why not all of the SVR genomes have elevated diversity in all genes with presumed interferon-suppressive functions. In some cases having suboptimal function in only one or two of the genes may have been sufficient to permit interferon α-based therapy to drive the HCV to extinction.

A novel association of higher viral diversity in NS2 with SVR was observed in genotype 1b. This association is difficult to interpret because although the NS2 protease function has been shown to be essential for assembly and production of infection virus [24]–[26]; there is no evidence for a direct role in modulation of the immune system. However, higher diversity in NS2 was also associated with null response compared to relapse in the genotype 1a NR sequences [11], and therefore NS2 appears to play a role in modulating the efficacy of antiviral therapy.

The genes in which high genetic diversity was associated with SVR were not the same in genotype 1a and 1b. This implies that HCV genotypes 1a and 1b do not rely on their common set of interferon-suppressive functions in the same pattern. However, the two genotypes respond to therapy at very similar rates [8], and there are no reported differences in the frequency with which they establish persistent infections. Therefore, although the mechanistic details of how the two subtypes counteract the type 1 interferon response appear to be somewhat different, the net efficacy of the mechanisms they employ seems to be similar.

Viral sequence differences are clearly not the only determinant of HCV's response to therapy. This was recently demonstrated by four groups who showed that single nucleotide polymorphisms (SNPs) in the IL28B gene are closely associated with SVR [27]–[30]. Suppiah *et. al.* [29] calculated that the population attributable risk for the favorable allele was 32%, indicating that variation within the IL28 locus is an important contributor to response, but is not the sole determining factor. Furthermore, the favorable allele at this locus is less frequent in African-Americans, partially explaining the poor response to treatment in the AA population [27], [30].

As an initial attempt to quantify the magnitude of the contribution of viral diversity to outcome of therapy, we asked if the number of variations unique to the early response categories (marked or poor) could quantitatively predict SVR. The probability of achieving SVR increased 2–3% with each additional unique variation in the polyprotein (Table 3). These data imply that a genotype 1a genome carrying 31 SVR-unique variations (the mean number in genotype 1 SVR sequences) would have a probability of response almost twice as high (31 variations X 3%/variation = 93%) than a 1a virus carrying the population consensus sequence that was used as a reference in this analysis. The effect size is similar to that reported for variation in the IL28B gene [27]. The location of the variations was important, because the quantitative effect of the number of variations was quite large for some proteins. This was especially notable in core, where there was a 26–28% increase in the probability of achieving SVR with each additional unique variation (Table 3). The magnitude of these quantitative effects was unexpectedly large given that the only way we could reduce noise from neutral genetic differences was to focus on variations unique to the SVR or NR groups.

The high proportion of AA patients enrolled in the Virahep-C study allowed us to assess the effect of HCV's genetic variation on the unusually poor response of AA patients to therapy. In genotype 1b, the viral diversity patterns associated with treatment outcome within the two racial groups were similar to the patterns observed when the race was disregarded (compare Figures 3D and 6B). The lower number of significant associations found when the racial groups were analyzed separately appears to be primarily due to the reduced statistical power from splitting the data set in half. However, in genotype 1a infected patients, the overall pattern of higher viral genetic diversity in the SVR patients that was present in the full patient set was exaggerated in the CA sequences, but diminished or absent in the AA sequences (compare Figures 3C and 6A). Therefore, the genotype 1a AA sequences largely lack the diversity patterns present in the 1a CA, 1b AA, and 1b CA sequences. The lack of genetic associations with SVR in the 1a AA patients implies that patient-specific factors may have been dominant over viral-specific variables in these patients in determining the outcome of therapy.

The SVR rate was 52% for CA and 28% for AA patients among the 201 participants in Virahep-C [8]. However, our viral genetics cohort of 94 Virahep-C patients was selected to contain equal numbers of day 28 responders and non-responders (the “marked” and “poor” groups) to adjust for response rate differences. This led to an over-sampling of the SVR patients in the AA group. The degree of over-sampling among the AA SVR patients was 28% [(AA_ sequenced SVR_)/AA_ total SVR_)/(CA_ sequenced SVR_/CA_ total SVR_) = 1.28]. The relative lack of viral genetic differences between HCV strains infecting the CA and AA patients strongly implies that the spectrum of viral strains circulating in the two racial groups is similar. In this context, the 28% over-sampling of the SVR patients in the AA population implies that although the viral strains in the CA and AA patients are similar, the AA population is infected with a higher proportion of relatively resistant viral strains. This preferential circulation of HCV strains in the AA population is plausible because AA patients are preferentially infected with genotype 1b compared to CA patients [31]. The 28% over-sampling ratio in the AA SVR patients therefore implies that one-quarter to one-third of the ∼2-fold difference in response rates for AA patients is due to preferential infection of the AA population with HCV strains that are relatively resistant to interferon α plus ribavirin therapy. This is consistent with recent work by Ge et. al. [27] which found that genetic variation in the IL28B gene was associated with the preferential clearance in CA patients, but that other factors were also involved in the discrepancy in treatment outcomes between CA and AA patients.

In sum, the presence of inter-patient HCV genetic variation associated with treatment efficacy that cannot be attributed to host selective pressures strongly implies that HCV genotype 1 sequences with varying sensitivity to interferon-based therapy circulate in the population. This does not exclude prominent roles for host factors in also affecting the outcome of therapy, such as genetic variation in the IL28B gene. However, the existence of viral genetic variations that are robustly associated with outcome of therapy raises the possibility that identification of key viral sequence motifs may help predict the outcome of therapy. The failures of predictive algorithms based on analysis of small regions of the genome such as the “interferon sensitivity determining region” [32], our own failure to find simple genetic lesions that sensitize the virus to therapy, and the distribution of diversity differences associated with treatment outcome across many viral genes indicate that identification of these viral motifs will require approaches that evaluate multiple viral genetic features. Furthermore, the different genetic associations with SVR in genotypes 1a and 1b indicate that such prognostic tests will need to be genotype-specific. Using this Virahep-C cohort we recently identified genome-wide amino acid covariance networks that were strikingly different in SVR and NR patients [33]. These networks (or other genome-wide analytical approaches) may be especially useful in identifying highly-resistant viral strains that would be non-responsive to interferon-based therapy. Infection with such strains would be a contraindication for therapy, eliminating the painful side-effects experienced by these patients during failed therapy while generating large financial savings in the health care system.

## Materials and Methods

### Virahep-C

Virahep-C was a study of peginterferon α and ribavirin therapy in treatment-naïve participants chronically infected with HCV genotype 1 [8]. Virahep-C enrolled 205 CA and 196 AA participants, all of whom were treated with peginterferon α-2a (Pegasys™, Roche Pharmaceuticals; 180 µg weekly by subcutaneous injection) and ribavirin (Copegus™, Roche Pharmaceuticals; 1000 mg/day for those who weighed <75 kg or 1200 mg/day for those who weighed ≥75 kg, orally). Treatment was for up to 48 weeks; therapy was discontinued for patients with detectable viremia at 24 weeks. Serum HCV RNA levels were quantified as described [8], and the primary outcome was SVR. All patients gave written informed consent to the Virahep-C study and its integral basic science studies, and this project was approved by the Saint Louis University Institutional Review Board.

### Sequencing

Consensus sequences for the full HCV ORF from pre-therapy samples (Genbank EF407411 to EF407504) were obtained by directly sequencing overlapping RT-PCR amplicons as described [12].

### Sequence Analyses

The genotype 1a and 1b samples were analyzed separately because sequence variation between the genotypes was anticipated to be larger than differences associated with response to therapy. All analyses except UU/UA frequency analyses and miRNA seed matches were conducted at the amino acid level. Sequence alignments were done with ClustalW. Positions that varied relative to the genotype 1a or 1b population consensus sequence were identified with Mutation Master [34]. The genotype 1a consensus reference sequence was derived from all 12 full-length ORFs in the Los Alamos [35] and European [36] HCV databases in April, 2005, plus five 1a ORFs we sequenced from non-Virahep-C cohorts. The genotype 1b population consensus reference sequence was generated from all 126 full-length ORFs from different patients in the databases in January, 2006. The known and predicted CD4^+^ and CD8^+^ T-cell epitope sequences were obtained from the HCV Immunology Database in July 2007 [16]. Shannon's entropy [37] was calculated with Bioedit [38]. The mean genetic distance was calculated using the p-distance algorithm in the MEGA v. 4 DNA analysis package [39].

### miRNA Seed Sequence Searches

The HCV sequences were searched for complimentarity to the seed sequences using Patmatch [40]. The seed sequences of each of the miRNAs were defined based on criteria described in [41].

### Statistical Analyses

Fisher's exact test was used to compare the proportions of unique variations between the response groups. Shannon's entropy values were compared using the Mann-Whitney rank-sums test, and the average genetic distances between the groups were compared using an independent samples t-test. The association of viral diversity and SVR was assessed through a Poisson (log-linear) regression model with a Bonferroni post-hoc correction. The number of unique variations between SVR and NR when split by race was compared using the Mann-Whitney rank-sums test. The level of significance (α) was set at 0.05 and statistical analyses were done using SAS software (SAS Institute, Inc.) or SPSS v. 13.0 (SPSS, Inc.).

## Supporting Information

Table S1 Comparison of the proportion of unique variations in the SVR and NR sequences.
(0.10 MB DOC)

Figure S1 Average genetic distance by treatment outcome. An alignment was created for the polyprotein and each individual protein (except p7). The p-distance was calculated for each pair in the alignment. The average genetic distance of the SVR sequences was compared to the NR sequences. The significance of the difference between the groups was determined using an independent samples t-test and is indicated for those genes where p≤<0.05. (A) Genotype 1a. (B) Genotype 1b.
(6.05 MB TIF)

Figure S2 Shannon's entropy of aligned sequences treatment outcome. An alignment was created for the polyproteins of each genotype. The entropy of each position in the alignment was calculated. The rank sum of the entropy for the SVR sequences was compared to the NR sequences for each protein. The significance of the difference between the groups was determined using an Mann-Whitney rank sums test and is indicated for those genes where p≤<0.05. (A) Genotype 1a. (B) Genotype 1b.
(5.57 MB TIF)

Figure S3 Frequency of miRNA seed matches to the HCV open reading frame between treatment outcome. The number of perfect matches for each class of miRNA was compared between SVR and NR sequences. The classes of miRNA were based on [17]. Those srepresented by the blue box are induced by interferonb but have no apprent anti-viral activity in culture. The miRNAs represented by the green boxes are induced by interferonb and have anti-viral activity towards HCV in culture. The miRNAs represented by the purple box are not induced by interferonb. miR-122 is a liver specific RNA that is required for HCV infection [18]. (A) Genotype 1a. (B) Genotype 1b.
(7.13 MB TIF)
