# Supplementary material for: Contribution of Genome-Wide HCV Genetic Differences to Outcome of Interferon-Based Therapy in Caucasian American and African American Patients
Source: PLoS One. 2010 Feb 3;5(2):e9032. doi: 10.1371/journal.pone.0009032 (PMC2815788; doi:10.1371/journal.pone.0009032)
Supplement: Table S1 — Comparison of the proportion of unique variations in the SVR and NR sequences. (0.10 MB DOC) [file pone.0009032.s001.doc]

**Table S1:** Comparison of the proportion of unique variations in the SVR and NR sequences.

| **Genotype 1a** | | | | |  | **Genotype 1b** | | | | |
| --- | --- | --- | --- | --- | --- | --- | --- | --- | --- | --- |
| **Polyprotein** | | | | | | | | | | |
|  | | SP^a^ | NS^b^ | Fisher’s Exact |  |  | | SP | NS | Fisher’s Exact |
| SVR | Count | 601 | 2,600 | **<0.001** |  | SVR | Count | 795 | 2,163 | **<0.001** |
|  | Exp^c^ | 501.7 | 2,699.3 |  |  |  | Exp | 667.5 | 2,290.5 |  |
| NR | Count | 413 | 2,855 |  |  | NR | Count | 363 | 1,811 |  |
|  | Exp | 512.3 | 2,755.7 |  |  |  | Exp | 490.5 | 1,683.5 |  |
| **Core** | | | | | | | | | | |
| SVR | Count | 18 | 8 | 0.052 |  | SVR | Count | 39 | 59 | **<0.001** |
|  | Exp | 15.0 | 11.0 |  |  |  | Exp | 27.9 | 70.1 |  |
| NR | Count | 5 | 9 |  |  | NR | Count | 4 | 49 |  |
|  | Exp | 8.1 | 6.0 |  |  |  | Exp | 15.1 | 37.9 |  |
| **E1** | | | | | | | | | | |
| SVR | Count | 65 | 141 | 0.013 |  | SVR | Count | 75 | 192 | 0.108 |
|  | Exp | 53.3 | 152.5 |  |  |  | Exp | 67.5 | 199.5 |  |
| NR | Count | 42 | 164 |  |  | NR | Count | 43 | 157 |  |
|  | Exp | 53.5 | 152.5 |  |  |  | Exp | 50.5 | 149.5 |  |
| **E2** | | | | | | | | | | |
| SVR | Count | 150 | 749 | 0.015 |  | SVR | Count | 210 | 678 | **<0.001** |
|  | Exp | 131.1 | 767.9 |  |  |  | Exp | 175.6 | 712.4 |  |
| NR | Count | 119 | 826 |  |  | NR | Count | 94 | 555 |  |
|  | Exp | 137.9 | 807.1 |  |  |  | Exp | 128.4 | 520.6 |  |
| **p7** | | | | | | | | | | |
| SVR | Count | 19 | 105 | 0.357 |  | SVR | Count | 16 | 35 | 1.000 |
|  | Exp | 14.8 | 109.2 |  |  |  | Exp | 15.8 | 35.2 |  |
| NR | Count | 10 | 109 |  |  | NR | Count | 10 | 23 |  |
|  | Exp | 14.2 | 104.8 |  |  |  | Exp | 10.2 | 22.8 |  |
| **NS2** | | | | | | | | | | |
| SVR | Count | 50 | 286 | 0.914 |  | SVR | Count | 98 | 289 | **<0.001** |
|  | Exp | 50.2 | 286.8 |  |  |  | Exp | 76.3 | 310.7 |  |
| NR | Count | 51 | 283 |  |  | NR | Count | 32 | 240 |  |
|  | Exp | 49.8 | 284.2 |  |  |  | Exp | 53.7 | 218.3 |  |
| **NS3/4A** | | | | | | | | | | |
| SVR | Count | 80 | 386 | **< 0.001** |  | SVR | Count | 111 | 260 | **<0.001** |
|  | Exp | 60.0 | 406.0 |  |  |  | Exp | 90.4 | 280.6 |  |
| NR | Count | 43 | 447 |  |  | NR | Count | 45 | 224 |  |
|  | Exp | 63.0 | 427.0 |  |  |  | Exp | 65.6 | 203.4 |  |
| **NS4B** | | | | | | | | | | |
| SVR | Count | 31 | 157 | 0.142 |  | SVR | Count | 37 | 65 | 0.025 |
|  | Exp | 26.2 | 161.8 |  |  |  | Exp | 30.0 | 72.0 |  |
| NR | Count | 22 | 170 |  |  | NR | Count | 18 | 67 |  |
|  | Exp | 26.8 | 165.2 |  |  |  | Exp | 25.0 | 60.0 |  |
|  | | | | | | | | | | |
|  | | | | | | | | | | |
|  | | | | | | | | | | |
|  | | | | | | | | | | |
|  | | | | | | | | | | |
| **NS5A** | | | | | | | | | | |
| SVR | Count | 132 | 486 | **<0.001** |  | SVR | Count | 143 | 325 | **<0.001** |
|  | Exp | 102.7 | 515.3 |  |  |  | Exp | 120.9 | 347.1 |  |
| NR | Count | 73 | 542 |  |  | NR | Count | 66 | 275 |  |
|  | Exp | 102.3 | 512.7 |  |  |  | Exp | 88.1 | 252.9 |  |
| **NS5B** | | | | | | | | | | |
| SVR | Count | 65 | 267 | 0.039 |  | SVR | Count | 66 | 260 | 0.680 |
|  | Exp | 54.8 | 277.2 |  |  |  | Exp | 63.8 | 262.2 |  |
| NR | Count | 48 | 305 |  |  | NR | Count | 51 | 221 |  |
|  | Exp | 58.2 | 294.8 |  |  |  | Exp | 53.2 | 218.8 |  |

^a^SP = Week 24 unique or specific variations

^b^NS = All variations or non-specific variations

^c^Exp = expected number of variations based on a chi-squared distribution.
