# Supplementary figures and images for: Contribution of Genome-Wide HCV Genetic Differences to Outcome of Interferon-Based Therapy in Caucasian American and African American Patients
Source: PLoS One. 2010 Feb 3;5(2):e9032. doi: 10.1371/journal.pone.0009032 (PMC2815788; doi:10.1371/journal.pone.0009032)

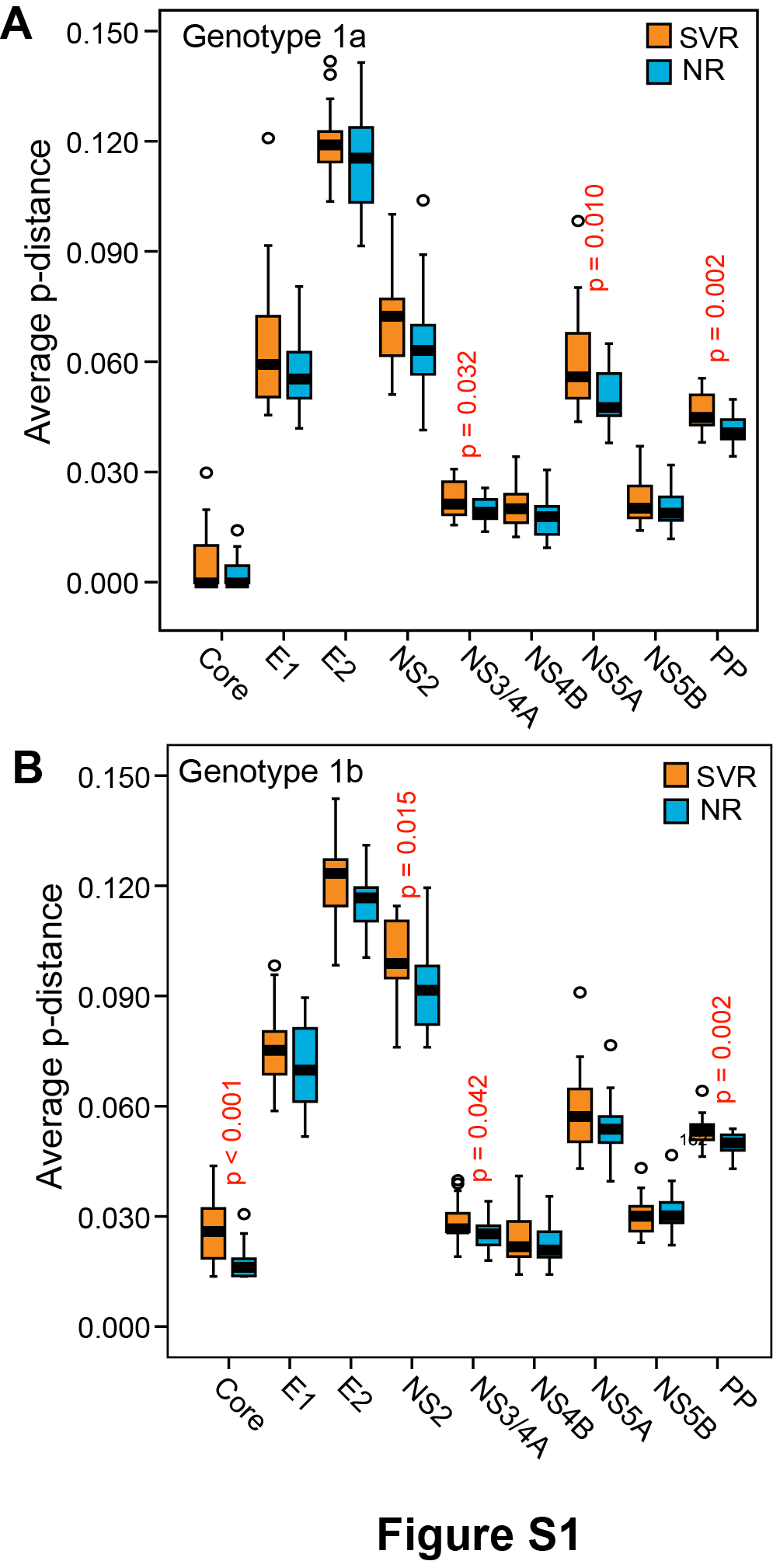

Supplement: Figure S1 — Average genetic distance by treatment outcome. An alignment was created for the polyprotein and each individual protein (except p7). The p-distance was calculated for each pair in the alignment. The average genetic distance of the SVR sequences was compared to the NR sequences. The significance of the difference between the groups was determined using an independent samples t-test and is indicated for those genes where p≤<0.05. (A) Genotype 1a. (B) Genotype 1b. (6.05 MB TIF) [file pone.0009032.s002.tif]

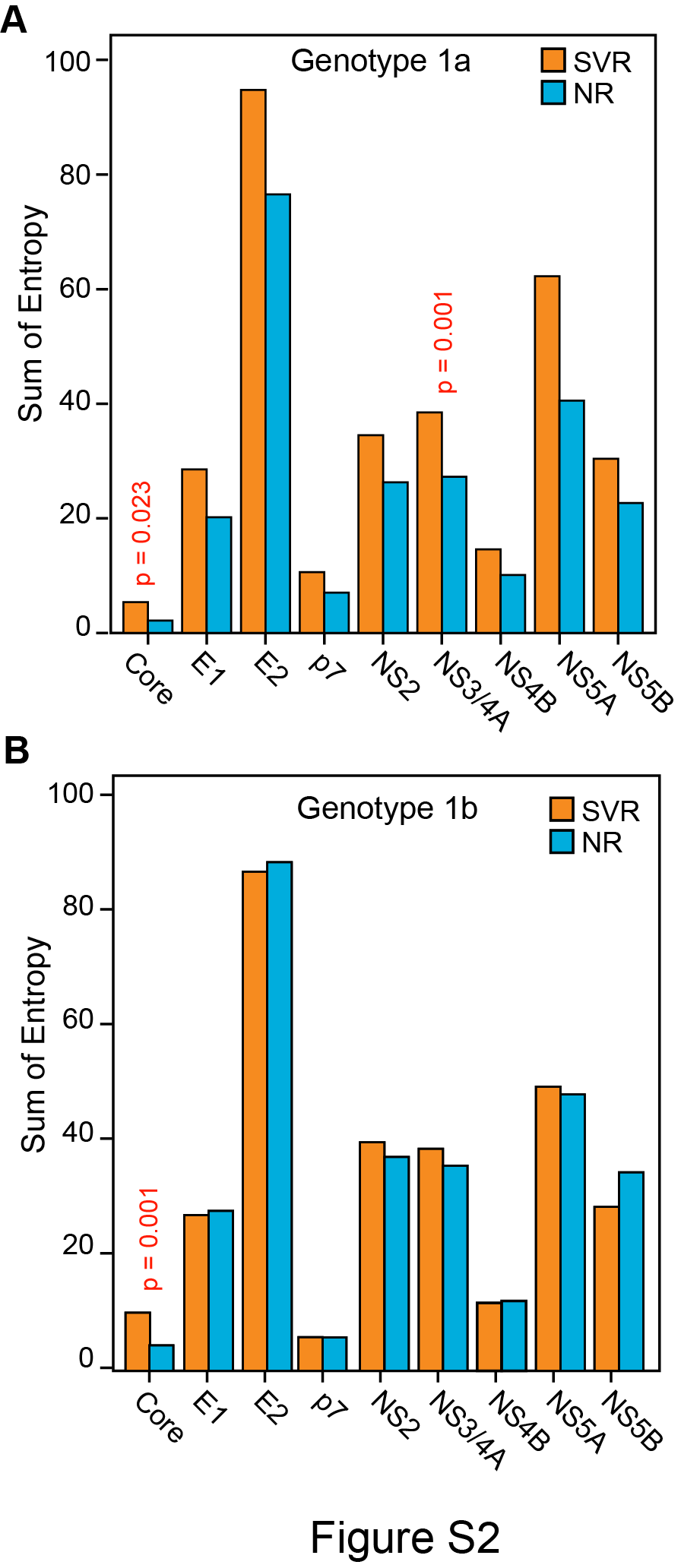

Supplement: Figure S2 — Shannon's entropy of aligned sequences treatment outcome. An alignment was created for the polyproteins of each genotype. The entropy of each position in the alignment was calculated. The rank sum of the entropy for the SVR sequences was compared to the NR sequences for each protein. The significance of the difference between the groups was determined using an Mann-Whitney rank sums test and is indicated for those genes where p≤<0.05. (A) Genotype 1a. (B) Genotype 1b. (5.57 MB TIF) [file pone.0009032.s003.tif]

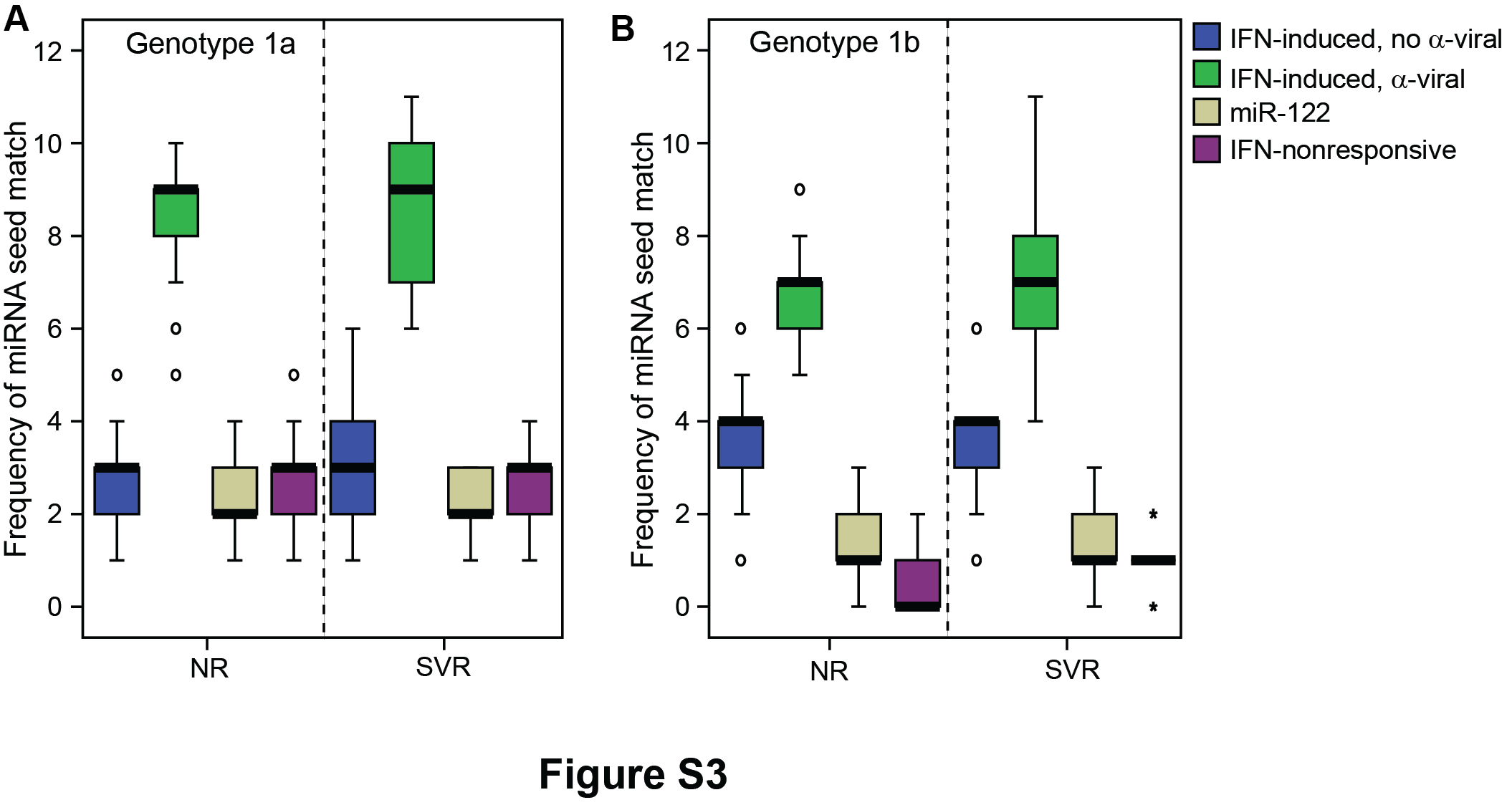

Supplement: Figure S3 — Frequency of miRNA seed matches to the HCV open reading frame between treatment outcome. The number of perfect matches for each class of miRNA was compared between SVR and NR sequences. The classes of miRNA were based on [17]. Those srepresented by the blue box are induced by interferonb but have no apprent anti-viral activity in culture. The miRNAs represented by the green boxes are induced by interferonb and have anti-viral activity towards HCV in culture. The miRNAs represented by the purple box are not induced by interferonb. miR-122 is a liver specific RNA that is required for HCV infection [18]. (A) Genotype 1a. (B) Genotype 1b. (7.13 MB TIF) [file pone.0009032.s004.tif]
